# Organ-Specific Phenolic Specialization and Antioxidant Responses in *Crocus cancellatus*: LC–MS/MS and Multivariate Analysis

**DOI:** 10.3390/ijms27198904

**Published:** 2026-10-07

**Authors:** Bedrettin Selvi

**Affiliations:** Department of Biology, Faculty of Arts and Sciences, Tokat Gaziosmanpaşa University, 60250 Tokat, Türkiye; bedrettin.selvi@gop.edu.tr; Tel.: +90-530-692-37-49

**Keywords:** *Crocus cancellatus*, phenolic compounds, organ-specific phytochemistry, LC–MS/MS, antioxidant capacity, hesperidin, multivariate analysis, principal component analysis

## Abstract

Organ-specific allocation of phenolic metabolites may shape antioxidant responses in *Crocus* species. This study characterized stamen, pistil, tepal, perigon tube, and corm of *Crocus cancellatus* Herb. using ultrasound-assisted methanolic extraction, liquid chromatography–tandem mass spectrometry with 30 authentic standards, six antioxidant assays, a relative antioxidant capacity index, Pearson correlation analysis, and principal component analysis. Twenty phenolic compounds were quantified. Hesperidin predominated in all organs and was highest in the tepal (17,660 ± 60 μg/g extract), approximately 43-fold above the corm. The pistil contained the highest 3-hydroxybenzoic acid (331 ± 1 μg/g extract) and 4-hydroxybenzoic acid (341 ± 2 μg/g extract), whereas the corm was enriched in pinoresinol (60.1 ± 0.8 μg/g extract) and uniquely contained vanillin (16.0 ± 0.6 μg/g extract). The tepal showed the highest total flavonoid content (81.50 ± 0.40 mg RE/g extract), while the stamen had the highest total phenolic content (12.63 ± 0.05 mg GAE/g extract). The corm showed the strongest DPPH radical scavenging (IC_50_ = 3.96 mg/mL), whereas the pistil led in ABTS and FRAP responses. Principal component analysis explained 64.16% of total variance and separated the five organ profiles. These findings demonstrate organ-specific phenolic specialization and support targeted bioactivity studies.

## 1. Introduction

The genus *Crocus* L. (Iridaceae) is a species-rich group distributed from the Mediterranean region and Europe eastward through western and Central Asia. Although *Crocus sativus* L., the source of saffron, has attracted the majority of phytochemical attention, the genus represents a diverse source of secondary metabolites, including apocarotenoids such as crocins, picrocrocin, and safranal, as well as flavonoids, flavonol glycosides, phenolic acids, and other specialized metabolites [1,2]. These compounds participate in several physiological and ecological processes, including pigmentation, UV protection, interactions with pollinators, defense against biotic stress, and protection against oxidative damage [3]. Their distribution within individual plants may vary substantially among anatomically and functionally distinct organs.

Organ-specific allocation of secondary metabolites is an important feature of plant chemical specialization. Floral tissues, particularly tepals and petals, frequently accumulate flavonoids and flavonol glycosides associated with pigmentation and UV-screening functions, whereas reproductive organs such as stamens and pistils can exhibit distinct phenolic compositions related to the physiological requirements of pollen- and stigma-associated tissues [1,3]. Underground storage organs such as corms may likewise accumulate distinct defensive and structural metabolites. Consequently, analyses of pooled or composite plant material may mask tissue-specific chemical differences, whereas organ-resolved investigations can reveal how specialized metabolites are partitioned among functionally distinct tissues.

Phytochemical investigations across *Crocus* species have revealed marked interspecific and organ-dependent variation in phenolic composition. In *C. sativus*, Mottaghipisheh et al. [1] analyzed tepal and stamen samples collected from 40 locations in Iran and identified kaempferol-3-*O*-sophoroside as the predominant tepal flavonol glycoside (62.19–99.48 mg/g), whereas substantially lower flavonoid concentrations were observed in stamens. Broader reviews of the genus have documented diverse phenolic acids and flavonoids across different *Crocus* taxa and plant parts [2]. Nevertheless, most phytochemical investigations have focused on *C. sativus* or have examined only one or two organs of non-saffron *Crocus* taxa, leaving systematic multi-organ comparisons comparatively scarce.

The taxonomy of the *Crocus cancellatus* complex has undergone substantial revision. In earlier treatments, several morphologically differentiated taxa were classified as subspecies of *C. cancellatus* [4], whereas subsequent taxonomic and phylogenetic studies have supported a narrower species-level treatment of several members of this complex [5]. Consistent with the current nomenclatural treatment adopted by Plants of the World Online and the Illustrated Flora of Turkey, the plant investigated in the present study is therefore referred to as *Crocus cancellatus* Herb. rather than *C. cancellatus* subsp. *cancellatus* [6].

Phytochemical information on *C. cancellatus* and taxa historically treated within the *C. cancellatus* complex remains limited. Loizzo et al. [7] investigated stigmas reported as *C. cancellatus* subsp. *damascenus*, identifying catechin hydrate, caffeic acid, and ferulic acid and evaluating several antioxidant and enzyme-inhibitory activities. Ismael [8] subsequently reported LC–MS/MS profiling of a species-level *C. cancellatus* ethanol extract and quantified ten compounds, including gallic, protocatechuic, ferulic, *p*-coumaric, and chlorogenic acids and quercetin. Shakeri et al. [9] applied LC-MS- and GC-MS-based metabolomics to stigmas reported as *C. cancellatus* subsp. *damascenus*, whereas Deniz et al. [10] characterized aerial and corm extracts reported as subsp. *mazziaricus* using HPLC and antioxidant assays. Alper et al. [11] evaluated total phenolic and flavonoid contents and antioxidant properties of aerial and bulb extracts reported as subsp. *lycuis*, without chromatographic profiling of individual phenolics. More recently, Özkan et al. [12] examined the phenolic composition and gastrointestinal bioaccessibility of corm material reported as subsp. *damascenus* using HPLC-PDA. Collectively, these studies demonstrate existing phytochemical interest in *C. cancellatus*-related taxa but also reveal the absence of a systematic organ-resolved quantitative LC–MS/MS comparison integrating multiple floral organs and the corm of *C. cancellatus*.

Reliable assessment of antioxidant capacity requires complementary assays because no single chemical method captures the full range of antioxidant mechanisms operating in complex plant extracts [13]. The phosphomolybdenum assay evaluates total reducing capacity through Mo(VI) reduction, DPPH and ABTS assays assess radical-scavenging responses, CUPRAC and FRAP measure cupric- and ferric-ion reducing capacity, respectively, and metal-chelating assays evaluate the capacity of extract constituents to bind transition-metal ions [14]. Because organ rankings may vary according to the chemical mechanism probed by each assay, integration of standardized assay responses using the relative antioxidant capacity index (RACI) can provide a complementary estimate of overall antioxidant performance [15].

Multivariate statistical approaches provide an additional means of integrating phytochemical composition with antioxidant responses. Pearson correlation analysis can identify linear associations between phenolic constituents and antioxidant variables, whereas hierarchical cluster analysis can reveal groups of variables displaying similar covariation patterns [16]. Principal component analysis (PCA) further reduces multidimensional datasets into orthogonal components, enabling visualization of sample separation and identification of variables contributing most strongly to organ differentiation. Together, these approaches provide an integrated framework for examining organ-specific relationships between phenolic composition and antioxidant responses.

We hypothesized that the distinct anatomical and physiological functions of the floral and underground organs of *C. cancellatus* are accompanied by organ-specific phenolic signatures associated with divergent antioxidant response patterns. To test this hypothesis, stamen, pistil, tepal, perigon tube, and corm extracts were comparatively characterized using quantitative LC–MS/MS phenolic profiling with 30 reference standards, total phenolic and flavonoid content determinations, six complementary antioxidant assays, RACI, Pearson correlation/HCA, and PCA. To the best of our knowledge, this is the first study to provide an organ-resolved quantitative LC–MS/MS phenolic characterization of *C. cancellatus* integrated with multivariate analysis of antioxidant responses.

## 2. Results

### 2.1. Total Phenolic and Flavonoid Contents and Relative Antioxidant Capacity Index

Total phenolic content (TPC), total flavonoid content (TFC), and relative antioxidant capacity index (RACI) values of the different organs are presented in Table 1. TPC ranged from 5.37 ± 0.28 mg GAE/g extract in the perigon tube to 12.63 ± 0.05 mg GAE/g extract in the stamen. The stamen, pistil (12.40 ± 0.19 mg GAE/g), and corm (11.71 ± 0.42 mg GAE/g) did not differ significantly from one another (*p* > 0.05), whereas the tepal (9.90 ± 0.19 mg GAE/g) and perigon tube exhibited significantly lower TPC values (*p* < 0.05).

TFC showed a markedly different organ-dependent pattern. The tepal exhibited the highest TFC (81.50 ± 0.40 mg RE/g extract), followed by the perigon tube (18.92 ± 0.34 mg RE/g), pistil (12.67 ± 0.19 mg RE/g), stamen (6.00 ± 2.94 mg RE/g), and corm (0.63 ± 0.01 mg RE/g). All five organs differed significantly from one another (*p* < 0.05). The TFC of the tepal was approximately 4.3-fold higher than that of the perigon tube and approximately 129-fold higher than that of the corm.

RACI values also differed among the organs. The pistil (0.52 ± 0.06) and stamen (0.38 ± 0.10) showed the highest RACI values and did not differ significantly from one another (*p* > 0.05). They were followed by the tepal (0.16 ± 0.07), corm (−0.08 ± 0.07), and perigon tube (−0.98 ± 0.03), which differed significantly from one another (*p* < 0.05).

### 2.2. LC–MS/MS Phenolic Profile of Different Plant Organs

**Figure 1:**
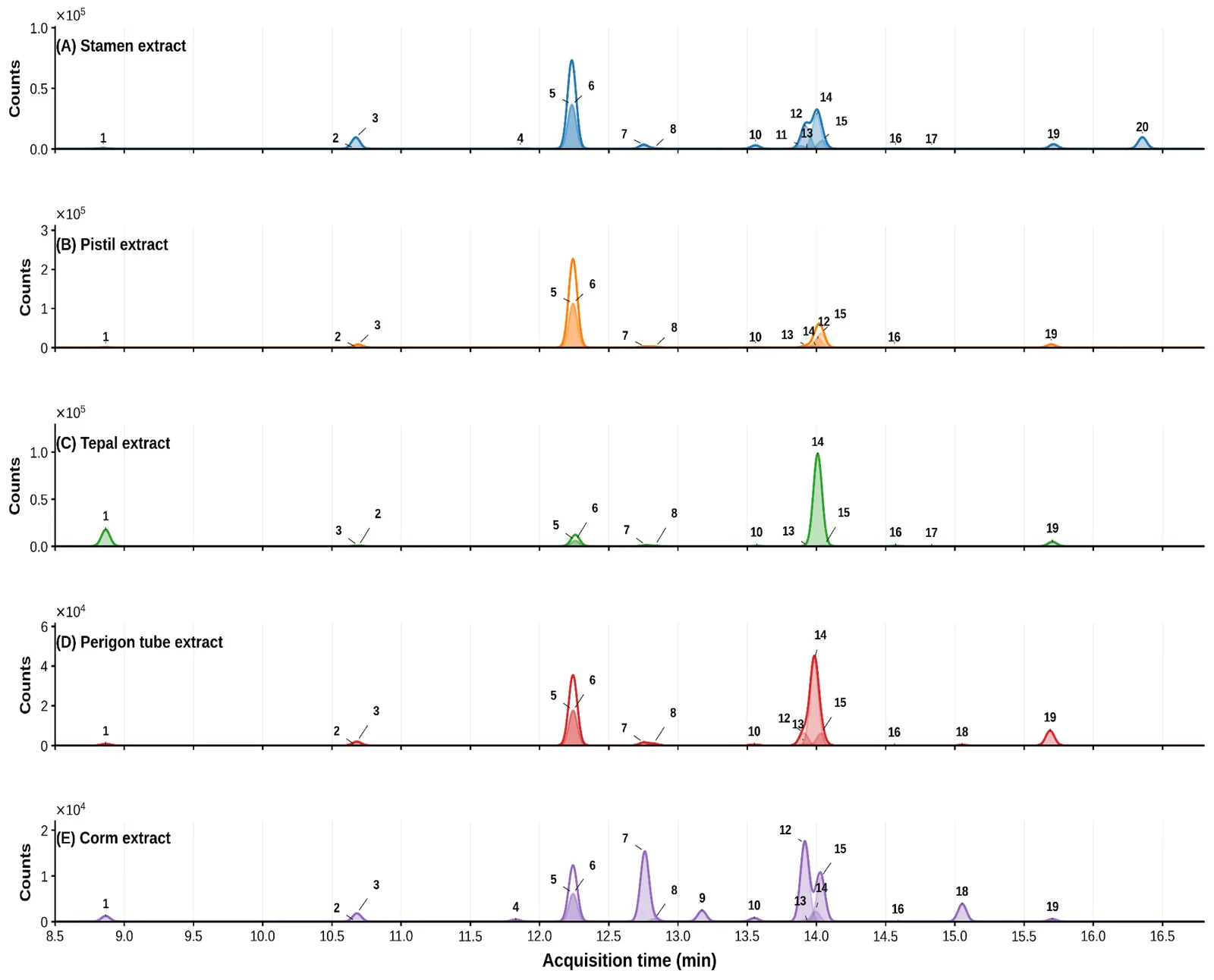
Representative LC–MS/MS chromatographic profiles of ultrasound-assisted methanolic extracts obtained from different organs of *C. cancellatus*: (**A**) stamen, (**B**) pistil, (**C**) tepal, (**D**) perigon tube, and (**E**) corm. The chromatograms display the MRM signals corresponding to the 20 target phenolic compounds detected in at least one of the five organ extracts; target compounds not detected in any organ were omitted from the figure. The x-axis represents acquisition time (min), and the y-axis represents counts. Peak numbers correspond to the following compounds: 1, gallic acid; 2, pyrocatechol; 3, protocatechuic acid; 4, chlorogenic acid; 5, 3-hydroxybenzoic acid; 6, 4-hydroxybenzoic acid; 7, caffeic acid; 8, syringic acid; 9, vanillin; 10, verbascoside; 11, taxifolin; 12, *p*-coumaric acid; 13, sinapic acid; 14, hesperidin; 15, ferulic acid; 16, hyperoside; 17, apigenin 7-glucoside; 18, pinoresinol; 19, quercetin; and 20, apigenin. Each organ extract is shown in a different color ((**A**), blue; (**B**), orange; (**C**), green; (**D**), red; (**E**), purple). Within each panel, the individual MRM traces of co-eluting compounds are superimposed as semi-transparent filled peaks, and leader lines connect peak numbers to the corresponding signals. Note the different y-axis scales (×10^5^ for (**A**–**C**) and ×10^4^ for (**D**,**E**)).

LC–MS/MS analysis screened 30 phenolic standards and detected 20 compounds across the five organs of *C. cancellatus*, while 10 compounds (luteolin 7-glucoside, 2,5-dihydroxybenzoic acid, 3,4-dihydroxyphenylacetic acid, (+)-catechin, (−)-epicatechin, rosmarinic acid, 2-hydroxycinnamic acid, eriodictyol, luteolin, and kaempferol) remained undetected in all organs (Table 2; Figure 1). The detected compounds included flavanone and flavonol glycosides, flavonoid aglycones, hydroxybenzoic and hydroxycinnamic acids, a catechol, a phenylethanoid glycoside, an aromatic aldehyde, and a lignan.

Hesperidin was the most abundant quantified compound in all five organs, although its concentration differed markedly among tissues. The highest concentration was observed in the tepal (17,660 ± 60 μg/g extract), followed by the perigon tube (8453 ± 27 μg/g), stamen (5479 ± 23 μg/g), pistil (1299 ± 8 μg/g), and corm (411 ± 6 μg/g) (*p* < 0.05). The tepal hesperidin concentration was approximately 43-fold higher than that of the corm. Hyperoside showed a broadly similar distribution, reaching its highest concentration in the tepal (286 ± 20 μg/g), although at substantially lower levels than hesperidin.

The pistil was characterized by the highest concentrations of 3-hydroxybenzoic acid (331 ± 1 μg/g) and 4-hydroxybenzoic acid (341 ± 2 μg/g), approximately threefold higher than those in the stamen and approximately 20-fold higher than those in the tepal. The pistil also contained the highest levels of ferulic acid (45.5 ± 0.1 μg/g), sinapic acid (14.8 ± 0.3 μg/g), quercetin (45.6 ± 0.3 μg/g), syringic acid (11.0 ± 0.3 μg/g), and *p*-coumaric acid (39.6 ± 0.7 μg/g) (Table 2).

The stamen contained the highest concentrations of protocatechuic acid (20.9 ± 0.5 μg/g), pyrocatechol (19.4 ± 0.5 μg/g), and verbascoside (8.53 ± 0.04 μg/g). Two compounds were detected exclusively in the stamen: apigenin (13.6 ± 0.4 μg/g) and taxifolin (2.96 ± 0.01 μg/g).

The corm showed a distinct phenolic distribution characterized by comparatively high levels of pinoresinol, caffeic acid, and vanillin. Pinoresinol accumulated predominantly in the corm (60.1 ± 0.8 μg/g) and was detected at a much lower concentration in the perigon tube (1.69 ± 0.08 μg/g). Caffeic acid was highest in the corm (21.7 ± 0.6 μg/g), approximately fivefold higher than in the stamen (4.16 ± 0.03 μg/g). Vanillin was detected exclusively in the corm (16.0 ± 0.6 μg/g), whereas chlorogenic acid was detected in the corm (4.10 ± 0.17 μg/g) and at a lower concentration in the stamen (2.00 ± 0.30 μg/g).

Gallic acid showed a distinct distribution, with the highest concentration in the tepal (81.1 ± 0.4 μg/g), approximately 17.5-fold higher than in the corm (4.63 ± 0.01 μg/g) and 43.6-fold higher than in the stamen (1.86 ± 0.02 μg/g). The perigon tube generally exhibited intermediate concentrations of several compounds; notably, it contained the second-highest concentrations of both hesperidin (8453 ± 27 μg/g) and quercetin (42.3 ± 0.1 μg/g).

The representative LC–MS/MS chromatographic profiles (Figure 1) were consistent with these organ-dependent quantitative patterns. The tepal profile was dominated by the hesperidin signal (peak 14), whereas the pistil showed prominent signals corresponding to 3-hydroxybenzoic acid (peak 5) and 4-hydroxybenzoic acid (peak 6). The corm displayed prominent signals for caffeic acid (peak 7) and *p*-coumaric acid (peak 12), together with a distinct pinoresinol signal (peak 18).

### 2.3. Antioxidant Activities

#### 2.3.1. Antioxidant Equivalent Values

Antioxidant activities expressed as Trolox equivalents (mg TE/g extract) or EDTA equivalents (mg EDTAE/g extract) are presented in Table 3. The antioxidant equivalent values varied among the five organs depending on the assay.

In the phosphomolybdenum assay, the tepal (394.93 ± 33.70 mg TE/g extract) and stamen (376.84 ± 29.33 mg TE/g extract) exhibited the highest values and did not differ significantly from one another (*p* > 0.05). Both showed significantly higher values than the pistil (279.31 ± 13.73 mg TE/g), corm (252.39 ± 10.61 mg TE/g), and perigon tube (195.47 ± 11.23 mg TE/g) (*p* < 0.05).

For DPPH radical scavenging, the corm showed the numerically highest equivalent value (35.38 ± 0.64 mg TE/g extract), followed by the tepal (29.87 ± 1.59 mg TE/g), pistil (26.55 ± 0.72 mg TE/g), perigon tube (15.12 ± 4.46 mg TE/g), and stamen (12.48 ± 0.24 mg TE/g). The corm and tepal did not differ significantly from one another (*p* > 0.05), whereas the corm exhibited a significantly higher value than the pistil, perigon tube, and stamen (*p* < 0.05).

ABTS radical scavenging activity was highest in the pistil (58.12 ± 0.06 mg TE/g extract), followed by the corm (46.17 ± 0.64 mg TE/g), tepal (39.00 ± 1.11 mg TE/g), stamen (35.29 ± 1.22 mg TE/g), and perigon tube (28.49 ± 0.70 mg TE/g). All five organs differed significantly from one another (*p* < 0.05).

CUPRAC reducing capacity was highest in the stamen (71.04 ± 1.44 mg TE/g extract), which exhibited a significantly higher value than all other organs (*p* < 0.05). The pistil (42.99 ± 2.20 mg TE/g), corm (37.60 ± 2.54 mg TE/g), and tepal (35.07 ± 3.37 mg TE/g) did not differ significantly from one another, whereas the perigon tube showed the lowest value (19.42 ± 3.23 mg TE/g).

FRAP reducing capacity was highest in the pistil (43.77 ± 0.32 mg TE/g extract), followed by the stamen (37.66 ± 0.89 mg TE/g), corm (28.65 ± 0.56 mg TE/g), perigon tube (21.81 ± 1.37 mg TE/g), and tepal (17.42 ± 0.16 mg TE/g). All five organs differed significantly from one another (*p* < 0.05).

Metal chelating activity varied within a narrow range, from 15.87 ± 0.14 mg EDTAE/g extract in the corm to 16.29 ± 0.08 mg EDTAE/g extract in the stamen. The stamen showed a significantly higher value than the corm (*p* < 0.05), whereas the pistil (16.21 ± 0.07 mg EDTAE/g), tepal (15.99 ± 0.10 mg EDTAE/g), and perigon tube (15.98 ± 0.06 mg EDTAE/g) did not differ significantly from either the stamen or corm (*p* > 0.05).

#### 2.3.2. IC_50_ and EC_0.5_ Values

The concentration-dependent activity parameters of the organ extracts are presented in Table 4. IC_50_ values were used for the DPPH and ABTS radical-scavenging assays and for metal-chelating activity, whereas EC_0.5_ values, defined as the concentrations required to produce an absorbance of 0.5, were used for the phosphomolybdenum, CUPRAC, and FRAP reducing-capacity assays. For both parameters, lower values indicate greater activity under the applied assay conditions.

In the phosphomolybdenum assay, the tepal (EC_0.5_ = 1.55 ± 0.13 mg/mL) and stamen (1.62 ± 0.13 mg/mL) showed the lowest EC_0.5_ values among the organ extracts, whereas the perigon tube showed the highest value (3.13 ± 0.18 mg/mL). For DPPH radical scavenging, the corm showed the lowest IC_50_ value (3.96 ± 0.07 mg/mL), followed by the tepal (4.69 ± 0.25 mg/mL), pistil (5.28 ± 0.14 mg/mL), perigon tube (9.68 ± 2.85 mg/mL), and stamen (11.22 ± 0.21 mg/mL). For ABTS radical scavenging, the lowest IC_50_ value was observed in the pistil (1.89 ± 0.01 mg/mL), followed by the corm (2.38 ± 0.03 mg/mL), tepal (2.82 ± 0.08 mg/mL), stamen (3.12 ± 0.11 mg/mL), and perigon tube (3.86 ± 0.09 mg/mL). For CUPRAC reducing capacity, the stamen showed the lowest EC_0.5_ value (1.32 ± 0.03 mg/mL), followed by the pistil (2.19 ± 0.11 mg/mL), corm (2.51 ± 0.17 mg/mL), tepal (2.69 ± 0.26 mg/mL), and perigon tube (4.91 ± 0.82 mg/mL). For FRAP reducing capacity, the pistil showed the lowest EC_0.5_ value (2.17 ± 0.02 mg/mL), followed by the stamen (2.52 ± 0.06 mg/mL), corm (3.32 ± 0.07 mg/mL), perigon tube (4.36 ± 0.27 mg/mL), and tepal (5.45 ± 0.05 mg/mL). Metal-chelating IC_50_ values varied within a narrow range of 1.04–1.07 mg/mL, with the lowest values observed for the stamen and pistil. Across all assays, the corresponding reference standards showed substantially lower IC_50_ or EC_0.5_ values than the organ extracts.

### 2.4. Pearson Correlation and Hierarchical Cluster Analysis

**Figure 2:**
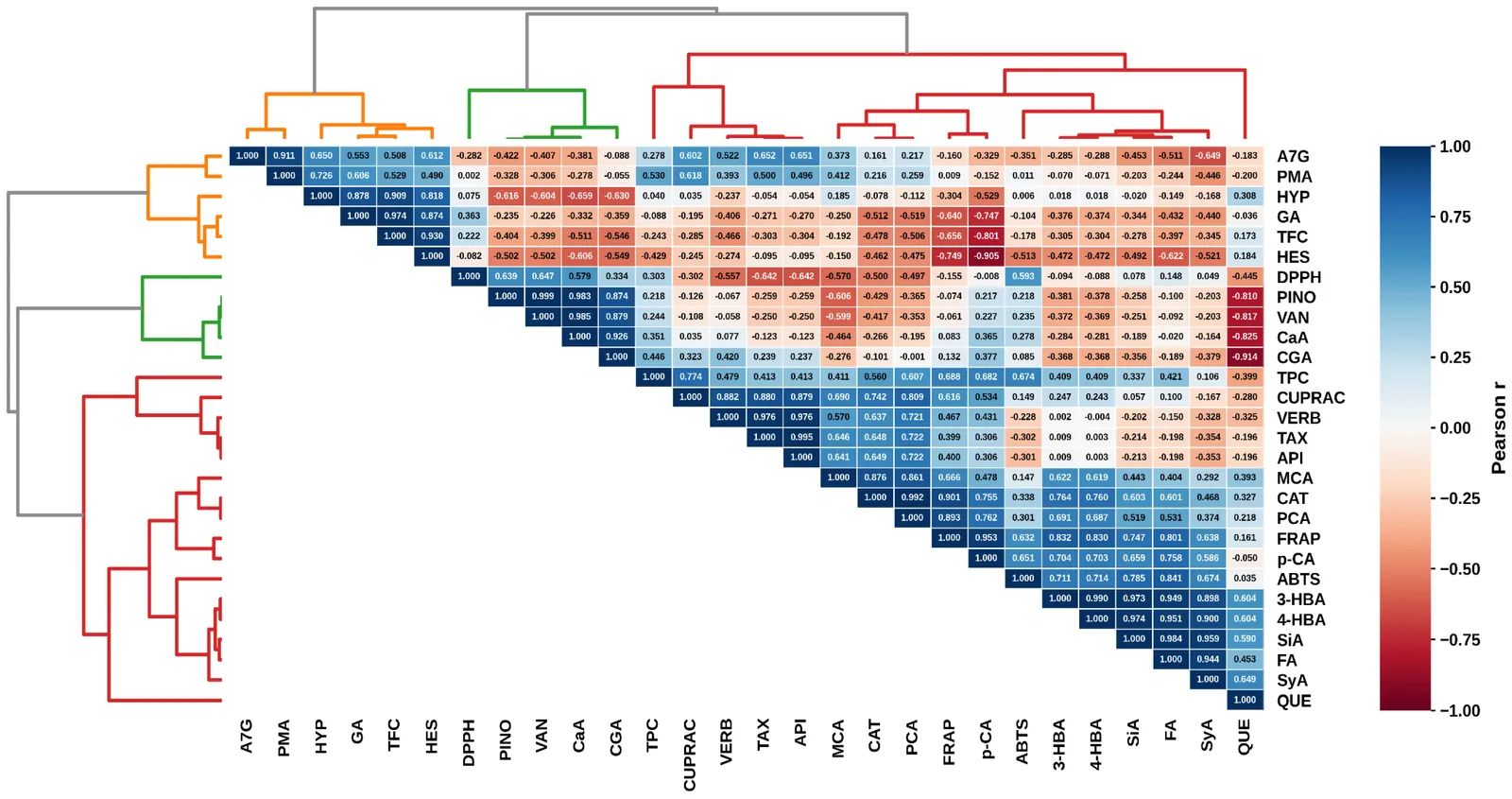
Hierarchically clustered Pearson correlation heatmap showing the relationships among antioxidant equivalent values, total phenolic and flavonoid contents, and individual phenolic compounds in ultrasound-assisted methanolic extracts obtained from different organs of *C. cancellatus*. Pearson correlation coefficients (r) are displayed within the cells, with positive correlations shown in blue and negative correlations in red. Hierarchical clustering was performed using the unweighted pair-group method with arithmetic mean (UPGMA; average linkage) based on a 1 − r distance matrix. The dendrograms on the top and left margins show the hierarchical clustering of variables; branch colors (orange, green, and red) denote the three main clusters, and gray branches indicate higher-level linkages joining these clusters. Only the upper triangular part of the correlation matrix, including the diagonal, is shown. The antioxidant variables included in the analysis were the equivalent values obtained from the phosphomolybdenum, DPPH, ABTS, CUPRAC, FRAP, and metal-chelating assays; IC_50_ and EC_0.5_ values were not included. Abbreviations: PMA, phosphomolybdenum assay; DPPH, 2,2-diphenyl-1-picrylhydrazyl radical scavenging activity; ABTS, 2,2′-azino-bis(3-ethylbenzothiazoline-6-sulfonic acid) radical scavenging activity; CUPRAC, cupric reducing antioxidant capacity; FRAP, ferric reducing antioxidant power; MCA, metal chelating activity; TFC, total flavonoid content; TPC, total phenolic content; HES, hesperidin; HYP, hyperoside; GA, gallic acid; QUE, quercetin; 3-HBA, 3-hydroxybenzoic acid; 4-HBA, 4-hydroxybenzoic acid; A7G, apigenin 7-glucoside; CAT, pyrocatechol; SyA, syringic acid; PCA, protocatechuic acid; VERB, verbascoside; CaA, caffeic acid; FA, ferulic acid; SiA, sinapic acid; API, apigenin; CGA, chlorogenic acid; VAN, vanillin; TAX, taxifolin; *p*-CA, *p*-coumaric acid; PINO, pinoresinol.

Pearson correlation analysis revealed distinct relationships among the 28 variables comprising antioxidant activities, TPC, TFC, and the 20 phenolic compounds detected in the five organ extracts (Figure 2). Among the phenolic constituents, 3-hydroxybenzoic acid (3-HBA) and 4-hydroxybenzoic acid (4-HBA) exhibited a near-perfect positive correlation (r = 0.990). Both compounds were also strongly positively correlated with sinapic acid (r = 0.973 and 0.974, respectively) and ferulic acid (r = 0.949 and 0.951, respectively). Within this phenolic acid group, sinapic acid was strongly correlated with ferulic acid (r = 0.984) and syringic acid (r = 0.959), while ferulic acid and syringic acid were also strongly correlated (r = 0.944).

FRAP showed positive correlations with 3-HBA (r = 0.832) and 4-HBA (r = 0.830), and its strongest association among the phenolic acids was observed with *p*-coumaric acid (r = 0.953). ABTS was positively correlated with 3-HBA (r = 0.711) and 4-HBA (r = 0.714), whereas the correlation between FRAP and ABTS was more moderate (r = 0.632).

A second set of strong relationships was observed among stamen-associated compounds. Pyrocatechol and protocatechuic acid were strongly correlated (r = 0.992). Verbascoside showed strong positive correlations with taxifolin and apigenin (r = 0.976 for both), while taxifolin and apigenin were almost perfectly correlated (r = 0.995). CUPRAC was positively correlated with verbascoside (r = 0.882), taxifolin (r = 0.880), and apigenin (r = 0.879), whereas its correlations with protocatechuic acid (r = 0.809) and pyrocatechol (r = 0.742) were comparatively lower. Protocatechuic acid and pyrocatechol were also positively correlated with FRAP (r = 0.893 and 0.901, respectively).

TFC was strongly positively correlated with gallic acid (r = 0.974), hesperidin (r = 0.930), and hyperoside (r = 0.909). TPC showed a positive correlation with CUPRAC (r = 0.774). Among the negative relationships, TFC was negatively correlated with *p*-coumaric acid (r = −0.801) and FRAP (r = −0.656), while hesperidin showed negative correlations with *p*-coumaric acid (r = −0.905) and FRAP (r = −0.749). Gallic acid was also negatively correlated with *p*-coumaric acid (r = −0.747). Apigenin 7-glucoside showed a strong positive correlation with phosphomolybdenum activity (r = 0.911).

Hierarchical cluster analysis using the UPGMA method separated the variables into three major clusters (Figure 2). The first cluster comprised A7G, PMA, HYP, GA, TFC, and HES, whereas the second comprised DPPH, PINO, VAN, CaA, and CGA. The third and largest cluster contained TPC, CUPRAC, VERB, TAX, API, MCA, CAT, PCA, FRAP, *p*-CA, ABTS, 3-HBA, 4-HBA, SiA, FA, SyA, and QUE. Within this cluster, particularly close relationships were evident for CAT–PCA, VERB–TAX–API, FRAP–*p*-CA, and 3-HBA–4-HBA.

### 2.5. Principal Component Analysis

**Figure 3:**
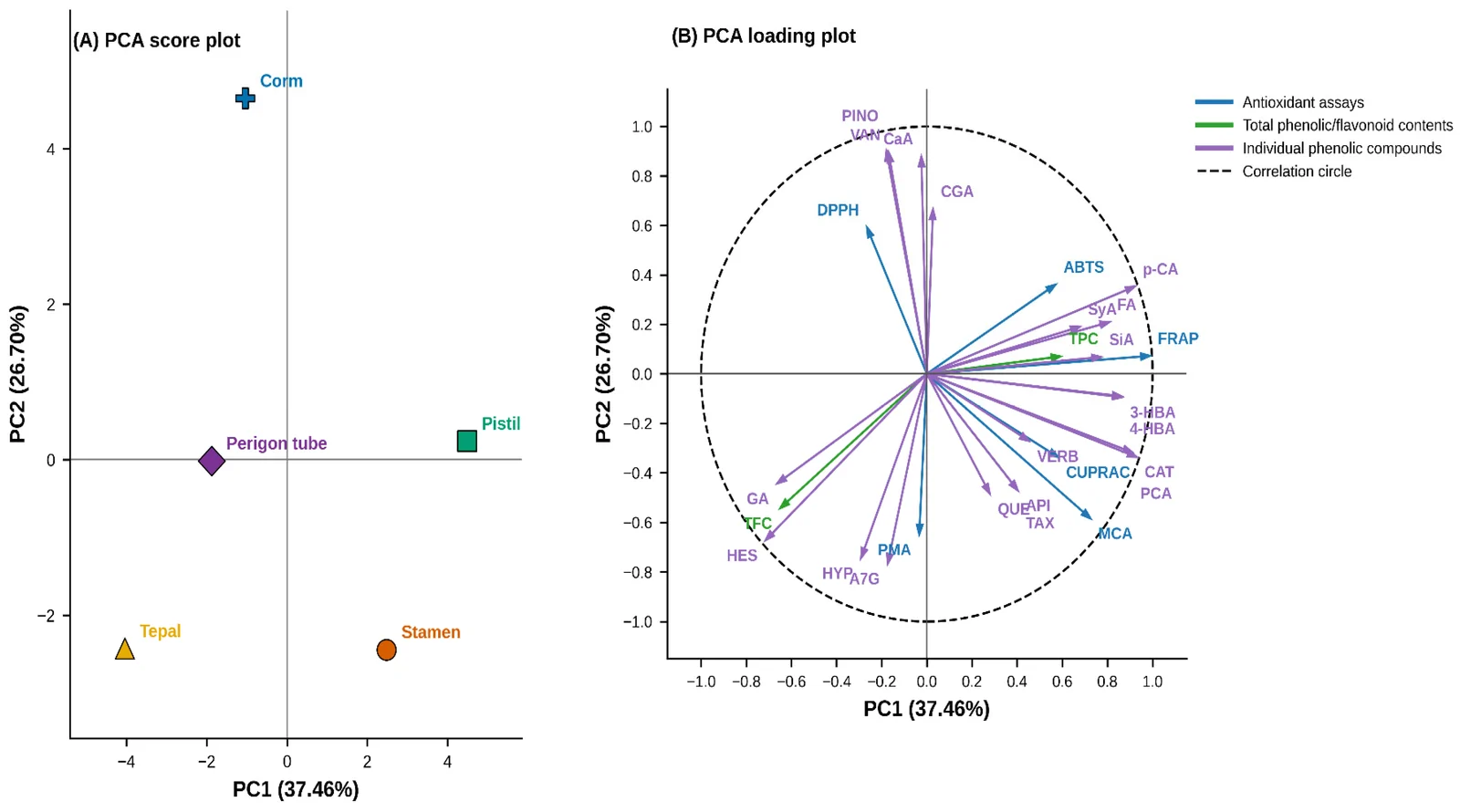
Principal component analysis (PCA) of phytochemical parameters, antioxidant activities, and identified phenolic compounds in ultrasound-assisted methanolic extracts obtained from different organs of *C. cancellatus*. (**A**) PCA score plot showing the distribution of stamen, pistil, tepal, perigon tube, and corm extracts, with each point representing the organ-level mean profile of the corresponding organ. (**B**) PCA loading plot based on the Pearson correlation matrix, illustrating the contribution and relationships of antioxidant assays, total phenolic and flavonoid contents, and individual phenolic compounds to the first two principal components. PC1 and PC2 explained 37.46% and 26.70% of the total variance, respectively, accounting for 64.16% of the cumulative variance. The dashed circle represents the correlation circle. Variables were color-coded according to their functional groups: antioxidant assays (blue), total phenolic and flavonoid contents (green), and individual phenolic compounds (purple). PMA, phosphomolybdenum assay; DPPH, 2,2-diphenyl-1-picrylhydrazyl radical scavenging activity; ABTS, 2,2′-azino-bis(3-ethylbenzothiazoline-6-sulfonic acid) radical scavenging activity; CUPRAC, cupric reducing antioxidant capacity; FRAP, ferric reducing antioxidant power; MCA, metal chelating activity; TFC, total flavonoid content; TPC, total phenolic content; HES, hesperidin; HYP, hyperoside; GA, gallic acid; QUE, quercetin; 3-HBA, 3-hydroxybenzoic acid; 4-HBA, 4-hydroxybenzoic acid; A7G, apigenin 7-glucoside; CAT, pyrocatechol; SyA, syringic acid; PCA, protocatechuic acid; VERB, verbascoside; CaA, caffeic acid; FA, ferulic acid; SiA, sinapic acid; API, apigenin; CGA, chlorogenic acid; VAN, vanillin; TAX, taxifolin; *p*-CA, *p*-coumaric acid; PINO, pinoresinol.

Principal component analysis (PCA) based on the Pearson correlation matrix showed that the first two principal components accounted for 64.16% of the total variance, with PC1 explaining 37.46% and PC2 explaining 26.70% (Figure 3).

The score plot placed the five organs in distinct regions of the PCA space (Figure 3A). The pistil and stamen were positioned on the positive side of PC1, although they differed along PC2, with the pistil showing a slightly positive PC2 score and the stamen a negative PC2 score. The tepal was located in the negative PC1/negative PC2 quadrant, whereas the corm occupied the negative PC1/positive PC2 quadrant. The perigon tube was positioned on the negative side of PC1 and close to the origin of PC2.

The loading plot revealed the variables contributing to these organ-associated patterns (Figure 3B). Positive PC1 loadings were observed for FRAP, 3-HBA, 4-HBA, sinapic acid, ferulic acid, syringic acid, *p*-coumaric acid, protocatechuic acid, pyrocatechol, CUPRAC, MCA, ABTS, and TPC. In contrast, TFC, hesperidin, gallic acid, and hyperoside showed negative PC1 loadings.

Along PC2, pinoresinol, vanillin, caffeic acid, chlorogenic acid, and DPPH showed positive loadings, corresponding to the position of the corm in the score plot. Negative PC2 loadings were observed for hyperoside, apigenin 7-glucoside, phosphomolybdenum activity, quercetin, apigenin, and taxifolin.

The orientations of several loading vectors were consistent with the Pearson correlation patterns described above. The FRAP and *p*-coumaric acid vectors showed similar orientations, consistent with their strong positive correlation (r = 0.953), whereas the TFC vector was oriented in the opposite direction to FRAP, in agreement with their negative association. The 3-HBA and 4-HBA vectors were nearly superimposed, reflecting their near-perfect positive correlation (r = 0.990). Pinoresinol and caffeic acid showed strong positive PC2 loadings and were positioned toward the corm-associated region of the PCA space.

Overall, the PCA revealed distinct organ-associated combinations of phenolic constituents and antioxidant variables, with the first two components capturing approximately two-thirds of the total variability in the dataset.

## 3. Discussion

### 3.1. Organ-Specific Phytochemical Specialization

The LC–MS/MS data revealed a pronounced organ-dependent distribution of phenolic constituents in *C. cancellatus*, with each organ exhibiting a distinct phytochemical profile. This pattern is consistent with the broader principle that specialized metabolite allocation may vary among anatomically and functionally distinct plant organs [3].

The tepal was distinguished by the marked accumulation of hesperidin (17,660 μg/g), together with elevated hyperoside (286 μg/g) and gallic acid (81.1 μg/g). This profile contrasts strongly with that reported for *C. sativus* tepals, in which kaempferol-3-*O*-sophoroside is the predominant flavonol glycoside (62.19–99.48 mg/g) [1]. Thus, the predominance of hesperidin in the present study indicates a markedly different tepal flavonoid composition in *C. cancellatus*. The exceptionally high TFC of the tepal (81.50 mg RE/g), approximately 129-fold higher than that of the corm, further supports the pronounced accumulation of flavonoid-related constituents in this floral organ.

The pistil showed a contrasting profile characterized by enrichment in hydroxybenzoic and hydroxycinnamic acids. It contained the highest concentrations of 3-hydroxybenzoic acid (331 μg/g), 4-hydroxybenzoic acid (341 μg/g), ferulic acid (45.5 μg/g), sinapic acid (14.8 μg/g), and *p*-coumaric acid (39.6 μg/g), together with the highest quercetin concentration (45.6 μg/g). To the best of our knowledge, such a pistil-specific phenolic profile has not previously been described for *C. cancellatus*-related taxa. The highly similar organ-level distribution patterns of 3-HBA and 4-HBA (r = 0.990) indicate strong co-variation across the five organs, although this association alone does not establish a shared biosynthetic origin.

The stamen was characterized by the highest concentrations of protocatechuic acid (20.9 μg/g), pyrocatechol (19.4 μg/g), and verbascoside (8.53 μg/g), together with the exclusive detection of apigenin (13.6 μg/g) and taxifolin (2.96 μg/g). Verbascoside (acteoside) is a widely distributed phenylethanoid glycoside with well-documented antioxidant and other biological activities [17]. To the best of our knowledge, its occurrence has not previously been reported in *C. cancellatus*. The combined enrichment of these compounds further differentiates the stamen from the other organs examined.

The corm exhibited a distinct profile characterized particularly by pinoresinol, caffeic acid, and vanillin. Pinoresinol accumulated predominantly in the corm (60.1 μg/g), although a much lower concentration was also detected in the perigon tube (1.69 μg/g). Caffeic acid reached its highest concentration in the corm (21.7 μg/g), approximately fivefold higher than in the stamen, whereas vanillin was detected exclusively in the corm (16.0 μg/g). Chlorogenic acid was also higher in the corm (4.10 μg/g) than in the stamen (2.00 μg/g), the only other organ in which it was detected. These findings establish the corm as chemically distinct without implying strict organ specificity for compounds that were also detected elsewhere.

Deniz et al. [10] reported ferulic acid (2376.2 μg/g) as the predominant phenolic in an ethanolic extract of *C. cancellatus* subsp. *mazziaricus*, whereas the corm examined in the present study contained 11.5 μg/g ferulic acid. However, because the HPLC phenolic profile reported by Deniz et al. [10] was not resolved separately for corm and aerial tissues, a direct organ-level quantitative comparison is not possible. Differences between the studies may additionally reflect taxonomic, environmental, extraction, and analytical factors.

The perigon tube displayed a profile sharing features with adjacent floral tissues, most notably its high hesperidin concentration (8453 μg/g) and the second-highest quercetin concentration (42.3 μg/g). Low-level pinoresinol (1.69 μg/g) was also detected in this organ. Together, these findings emphasize that phenolic constituents are distributed non-uniformly among the five anatomically distinct organs of *C. cancellatus*.

### 3.2. Relationships Between Phenolic/Flavonoid Contents and Antioxidant Responses

The contrasting patterns of TPC, TFC, and individual antioxidant assays indicate that total phenolic and flavonoid contents do not predict all dimensions of antioxidant response equally. The tepal, despite exhibiting the highest TFC (81.50 mg RE/g) and a TPC of 9.90 mg GAE/g, did not lead the FRAP, ABTS, or CUPRAC assays but showed comparatively strong performance in the phosphomolybdenum and DPPH assays. In contrast, the stamen and pistil, which showed the numerically highest TPC values (12.63 and 12.40 mg GAE/g, respectively), exhibited the highest CUPRAC and FRAP capacities, although their TPC values did not differ significantly from those of the corm. Such assay-dependent differences are expected because antioxidant methods probe distinct reaction mechanisms and may respond differently to individual phenolic structures and complex extract compositions [14].

The tepal profile provides a clear example of this distinction. Its phenolic composition was dominated by hesperidin, together with elevated hyperoside and gallic acid. Flavonoid antioxidant behavior is strongly influenced by structural features such as hydroxylation pattern, substitution, and glycosylation; in particular, ortho-dihydroxyl arrangements are important determinants of electron- and hydrogen-donating capacity [18]. Thus, the predominance of hesperidin may partly contribute to the assay-dependent behavior of the tepal, although the present correlations do not establish a direct mechanistic contribution of this individual compound.

The pistil, by contrast, was enriched in hydroxybenzoic and hydroxycinnamic acids. The organ-level distributions of 3-HBA and 4-HBA were positively associated with FRAP (r = 0.832 and 0.830, respectively) and ABTS (r = 0.711 and 0.714, respectively), while *p*-coumaric acid showed a particularly strong positive correlation with FRAP (r = 0.953). Phenolic acids can display markedly different reducing and radical-scavenging properties depending on the number and position of hydroxyl and other substituent groups [14,18]. These relationships are therefore consistent with the distinct phenolic acid-rich profile of the pistil and its high FRAP and ABTS responses, although they should be interpreted as associations rather than evidence that individual compounds directly determine extract-level antioxidant activity. The observed antioxidant responses may additionally reflect contributions from unquantified constituents and potential additive, synergistic, or antagonistic interactions within the complex extract matrix [19].

The stamen showed a different chemical–antioxidant pattern. Protocatechuic acid and pyrocatechol, both enriched in this organ, contain ortho-dihydroxyl arrangements that are generally favorable for electron donation and redox activity [14,18]. Their correlations with CUPRAC were positive (r = 0.809 and 0.742, respectively); however, stronger CUPRAC associations were observed for verbascoside (r = 0.882), taxifolin (r = 0.880), and apigenin (r = 0.879). The high CUPRAC response of the stamen therefore appears to be associated with a constellation of co-occurring phenolic constituents rather than with a single compound.

At the aggregate level, TPC was positively correlated with CUPRAC (r = 0.774), indicating similar organ-level variation in these parameters. In contrast, TFC was negatively correlated with FRAP (r = −0.656) and *p*-coumaric acid (r = −0.801), while hesperidin showed stronger negative associations with FRAP (r = −0.749) and *p*-coumaric acid (r = −0.905). These inverse relationships primarily reflect the contrasting organ-specific profiles of the flavonoid-rich tepal and the phenolic acid-enriched pistil and should not be interpreted as evidence of direct antagonistic effects. Overall, the findings underscore the importance of phenolic composition, rather than total phenolic or flavonoid content alone, when interpreting assay-dependent antioxidant responses in complex plant extracts [14].

### 3.3. Assay-Dependent Antioxidant Responses

The divergent organ rankings observed across the antioxidant assays emphasize that antioxidant response is assay-dependent and reflects the different chemical mechanisms probed by each method [14]. Consequently, no single organ consistently exhibited the highest response across all assays, highlighting the importance of interpreting complementary antioxidant methods together rather than relying on a single measurement.

In the phosphomolybdenum assay, the tepal and stamen showed the highest equivalent values and did not differ significantly from one another. Apigenin 7-glucoside was positively correlated with phosphomolybdenum activity (r = 0.911); however, this association primarily reflects similar organ-level variation and does not establish a direct contribution of this compound to Mo(VI) reduction.

The corm showed the numerically strongest DPPH response, with the highest equivalent value (35.38 mg TE/g) and the lowest IC_50_ value (3.96 mg/mL), although it did not differ significantly from the tepal in equivalent capacity or from the tepal and pistil in IC_50_. The corm was also characterized by enrichment in pinoresinol and caffeic acid. In the PCA loading plot, DPPH, pinoresinol, and caffeic acid showed positive PC2 loadings toward the corm-associated region, indicating co-variation among these variables rather than a demonstrated mechanistic contribution of individual compounds to DPPH scavenging.

The pistil exhibited the highest ABTS equivalent value (58.12 mg TE/g) and FRAP reducing capacity (43.77 mg TE/g). This pattern was accompanied by its enrichment in hydroxybenzoic and hydroxycinnamic acids. In particular, 3-HBA and 4-HBA were positively associated with FRAP (r = 0.832 and 0.830, respectively), while *p*-coumaric acid showed a stronger correlation with FRAP (r = 0.953). By contrast, the correlation between FRAP and ABTS was more moderate (r = 0.632), indicating that the high responses of the pistil in these assays should not be interpreted as evidence that both assays are governed by an identical set of constituents.

The stamen showed the highest CUPRAC reducing capacity (71.04 mg TE/g), significantly exceeding the other organ extracts. This response coincided with a distinct phenolic profile containing protocatechuic acid, pyrocatechol, verbascoside, apigenin, and taxifolin. As indicated by the correlation analysis, the CUPRAC response was associated with several co-occurring constituents rather than with a single phenolic compound.

Metal-chelating activity varied only slightly among organs (15.87–16.29 mg EDTAE/g), with a significant difference observed between the stamen and corm but substantial statistical overlap among the remaining organs. The narrow range of values indicates that metal-chelating capacity provided comparatively limited discrimination among the five organ extracts.

RACI placed the pistil (0.52) and stamen (0.38) in the highest statistical group, whereas the perigon tube showed the lowest value (−0.98). As a composite index, RACI provides an integrated view of the measured parameters but should be interpreted together with the individual antioxidant assays rather than as a substitute for assay-specific information.

Comparison with previous studies further illustrates the strong influence of experimental design and plant material on reported antioxidant values. Alper et al. [11] reported DPPH IC_50_ values of 0.86 ± 0.03 mg/mL for aerial-part and 1.11 ± 0.01 mg/mL for bulb methanolic extracts of *C. cancellatus* subsp. *lycuis*, both lower than the values observed for the individual organs examined here. However, their aerial-part material was not resolved into separate organs, and their methanolic extraction conditions differed substantially from the ultrasound-assisted procedure used in the present study, limiting direct quantitative comparison. Loizzo et al. [7] reported a substantially lower DPPH IC_50_ value of 34.6 μg/mL for an isolated stigma extract reported as *C. cancellatus* subsp. *damascenus*. Because the stigma was not analyzed separately in the present study and the extraction and assay conditions differed, these values should be regarded as contextual rather than directly comparable measurements.

### 3.4. Multivariate Integration of Metabolite and Antioxidant Data

The combined Pearson/HCA and PCA analyses provided an integrated view of the organ-dependent relationships among phenolic composition and antioxidant responses. Rather than identifying direct causal relationships, these multivariate approaches revealed groups of variables displaying similar patterns of variation across the five organs and highlighted the major dimensions underlying organ differentiation [16].

Pearson analysis identified several strong patterns of co-variation among organ-enriched phenolic constituents. The particularly strong associations among 3-HBA, 4-HBA, sinapic acid, and ferulic acid were consistent with their preferential accumulation in the pistil, while 3-HBA and 4-HBA also showed positive associations with FRAP (r = 0.832 and 0.830, respectively). Notably, *p*-coumaric acid showed a strong positive correlation with FRAP (r = 0.953). In contrast, the flavonoid-rich tepal profile was characterized by strong positive associations among TFC, hesperidin, hyperoside, and gallic acid. These relationships primarily reflect organ-level co-variation and should not be interpreted as evidence that individual compounds directly determine the corresponding antioxidant responses. Because the extracts represent chemically complex mixtures, the measured activities may also involve unquantified constituents and interactions among multiple components; therefore, the present correlation analysis cannot distinguish the contribution of individual compounds from potential combination effects [19].

The HCA supported this organ-associated organization by separating the variables into three major clusters. The first cluster comprised A7G, PMA, HYP, GA, TFC, and HES and was largely associated with the tepal-related flavonoid pattern. A second cluster consisting of DPPH, PINO, VAN, CaA, and CGA corresponded closely to the corm-associated chemical profile. The third and largest cluster included TPC, CUPRAC, VERB, TAX, API, MCA, CAT, PCA, FRAP, *p*-CA, ABTS, 3-HBA, 4-HBA, SiA, FA, SyA, and QUE, integrating variables associated predominantly with the pistil- and stamen-related profiles. These clusters represent similarities in cross-organ variation rather than demonstrated biosynthetic co-regulation.

PCA further summarized these relationships into two principal dimensions accounting for 64.16% of the total variance. PC1 (37.46%) placed the pistil and stamen on the positive side and the tepal, perigon tube, and corm on the negative side. Positive PC1 loadings were associated mainly with phenolic acids, TPC, and reducing/antioxidant variables including FRAP and CUPRAC, whereas TFC, hesperidin, hyperoside, and gallic acid loaded negatively. Thus, PC1 primarily represented a phenolic-acid/reducing-response versus flavonoid/gallic-acid gradient across the organ profiles.

PC2 (26.70%) further distinguished the corm, which showed a strong positive score, from particularly the stamen and tepal on the negative side. Positive PC2 loadings included pinoresinol, vanillin, caffeic acid, chlorogenic acid, and DPPH, whereas apigenin 7-glucoside, phosphomolybdenum activity, hyperoside, apigenin, taxifolin, and quercetin contributed in the negative direction. This pattern highlights a corm-associated dimension characterized by lignan/hydroxycinnamic-acid enrichment and DPPH response.

The orientation of the loading vectors was consistent with several of the Pearson relationships. In particular, the FRAP and *p*-coumaric acid vectors showed similar orientations, in agreement with their strong positive correlation (r = 0.953). Likewise, the near-superposition of the 3-HBA and 4-HBA vectors reflected their highly similar distributions across organs (r = 0.990), representing one of the strongest patterns of metabolite co-variation in the dataset.

Comparable multivariate approaches have also been used to characterize organ- and population-dependent phytochemical variation in *Crocus*. Mottaghipisheh et al. [1] applied PCA to flavonol profiles of *C. sativus* tepal and stamen samples collected from 40 Iranian locations and reported that four principal components accounted for 89.17% of the total variation, with PC1 showing a particularly strong association with stamen quercetin-3-*O*-sophoroside. Direct comparison of cumulative variance with the present two-component model is not appropriate because the two studies differ substantially in sample structure, number and type of variables, and number of retained principal components. Nevertheless, both studies illustrate the utility of multivariate analysis for resolving phytochemical differentiation within *Crocus*.

### 3.5. Scientific Significance, Limitations, and Future Directions

To the best of our knowledge, this study provides the first organ-resolved quantitative LC–MS/MS characterization of phenolic constituents in *C. cancellatus* across five anatomically distinct organs. The identification of distinct organ-associated profiles—including hesperidin predominance in the tepal, hydroxybenzoic and hydroxycinnamic acid enrichment in the pistil, protocatechuic acid/pyrocatechol/verbascoside enrichment in the stamen, and pinoresinol/caffeic acid enrichment in the corm—provides a phytochemical framework for further investigation of phenolic allocation in this taxon.

Several limitations should be considered when interpreting these findings. First, only methanolic extraction was employed; consequently, the resulting profile may favor compounds with particular polarity and solubility characteristics and should not be considered exhaustive. Second, the targeted LC–MS/MS analysis was restricted to the 30 reference compounds included in the analytical panel, and phenolic constituents outside this panel were therefore not assessed. Third, the antioxidant measurements were based exclusively on in vitro chemical assays. Although these methods provide complementary information on radical scavenging, reducing capacity, and metal chelation, they do not assess cellular uptake, metabolism, bioavailability, or in vivo antioxidant effects.

The multivariate associations also require cautious interpretation. The observed correlations between individual phenolic compounds and antioxidant variables indicate patterns of co-variation across organ profiles but do not demonstrate direct causal contributions of specific compounds to extract-level activity. The measured responses may additionally reflect the combined effects of multiple detected and unmeasured constituents and their potential interactions. Moreover, as currently described, spectrophotometric measurements were performed in technical triplicate; such replication primarily estimates analytical precision and does not by itself capture biological variability among independent plants or populations.

A further limitation is that the plant material originated from a single locality and collection event. Phenolic composition may vary with population, altitude, developmental stage, seasonal conditions, and interannual environmental variation. Accordingly, the organ-specific profiles reported here should be regarded as a quantitative baseline for the analyzed population and collection period rather than as invariant chemical characteristics of the taxon.

These findings provide several directions for future research. Validation of the identified organ-associated signatures across independent populations, seasons, and developmental stages will be necessary to determine their reproducibility. Broader targeted panels or untargeted metabolomic approaches could reveal additional constituents not captured by the present 30-compound LC–MS/MS panel. Isolation and individual testing of selected compounds, together with experiments using defined compound combinations, could help distinguish individual from interactive contributions to extract-level antioxidant responses. Finally, evaluation of the organ-specific extracts and selected constituents in cellular oxidative-stress models would provide an important step toward determining whether the chemical antioxidant responses observed here translate into biological effects.

## 4. Materials and Methods

### 4.1. Plant Material

Plant material of *Crocus cancellatus* Herb. was collected on 17 September 2024 from the Batanca locality near Göçü Village, Beyşehir district, Konya Province, Türkiye (37°45′16″ N, 31°47′42″ E; 1175 m a.s.l.), where the plants were growing beneath a pine forest. Sufficient plant material was collected to ensure that at least 5.0 g of dried material was available for each organ-specific pooled composite sample. Immediately after collection, the plants were separated into five anatomical parts: stamen, pistil (stigma, style, and ovary), tepal, perigon tube, and corm. For each organ, the corresponding material obtained from the collected plants was pooled to obtain a single composite sample.

The plant material was morphologically identified as *Crocus cancellatus* Herb. by Dr. Bedrettin Selvi based on established taxonomic treatments [4,6]. Identification was based on a combination of diagnostic morphological characters, including an ovoid–pyriform corm with coarsely reticulate-fibrous outer tunics and smoother, papery inner layers; autumn flowering with hysteranthous leaves bearing a white median stripe; absence of a prophyll; a bracteole concealed within and markedly shorter than the bract; pale lilac to whitish, darker-veined tepals with a glabrous, white to pale-yellow throat; yellow anthers and glabrous filaments; and a style divided into numerous slender orange branches that usually overtop the anthers. The concealed bracteole distinguishes the examined material from *C. mazziaricus*, *C. lycius*, and *C. pamphylicus*, in which the bracteoles are exserted, whereas the ovoid–pyriform corm and layered tunic structure distinguish it from *C. damascenus*, which is characterized by a subglobose corm with uniformly coarse, woody tunics. A voucher specimen (GOPU 9225) was deposited in the Herbarium of the Faculty of Arts and Sciences, Tokat Gaziosmanpaşa University.

### 4.2. Preparation of Methanolic Extracts

Methanolic extracts were prepared using ultrasound-assisted extraction (UAE) based on previously reported procedures [20,21]. For each organ, 5.0 g of the powdered composite sample was mixed with 100 mL of HPLC-grade methanol at a solid-to-solvent ratio of 1:20 (*w*/*v*). Extraction was performed once for each organ in an ultrasonic bath operating at 40 kHz and 260 W, with the bath temperature maintained at approximately 30 °C for 60 min. Following sonication, the mixtures were filtered through Whatman No. 1 filter paper, and the solvent was removed under reduced pressure using a rotary evaporator at 40 °C.

The resulting extracts were redissolved in methanol to prepare stock solutions at a concentration of 15 mg/mL. Appropriate dilutions of these stock solutions were prepared according to the concentration ranges required for the individual phytochemical and antioxidant assays. Extract solutions were stored at 4 °C until analysis.

Extraction yield was calculated on a dry-weight basis according to the equation: extraction yield (%) = (dry extract weight/dry starting material weight) × 100. The extraction yields were 57.11%, 43.18%, 38.72%, 23.88%, and 6.99% (*w*/*w*) for the stamen, pistil, tepal, perigon tube, and corm, respectively. The comparatively low extraction yield of the corm may partly reflect its distinct storage-organ matrix, as *Crocus* corms accumulate substantial starch reserves [22], which are poorly represented in methanol-soluble plant fractions [23]; however, this matrix-related explanation remains tentative because corm carbohydrate composition was not directly determined in the present study.

### 4.3. Phytochemical Analysis

#### 4.3.1. Determination of Total Phenolic and Flavonoid Contents

Total phenolic content (TPC) was determined using the Folin–Ciocalteu method and expressed as milligrams of gallic acid equivalents per gram of extract (mg GAE/g extract), whereas total flavonoid content (TFC) was determined using the aluminium chloride colorimetric method and expressed as milligrams of rutin equivalents per gram of extract (mg RE/g extract), following previously reported procedures with minor modifications [24,25]. Detailed reagent volumes, incubation conditions, and spectrophotometric procedures are provided in the Supplementary Materials.

#### 4.3.2. LC–ESI–MS/MS Analysis of Phenolic Compounds

Targeted phenolic analysis was performed using an LC–ESI–MS/MS method adapted from the previously validated procedure reported by Cittan and Çelik [26]. Thirty phenolic compounds were included in the targeted analytical panel. Quantitative analyses were performed using an Agilent Technologies 1260 Infinity liquid chromatography system coupled to an Agilent 6420 Triple Quadrupole mass spectrometer. Chromatographic separation was achieved on a Poroshell 120 EC-C18 column (100 mm × 4.6 mm i.d., 2.7 μm particle size).

The mobile phase consisted of solvent A (0.1% *v*/*v* formic acid in water) and solvent B (methanol), using the following gradient program: 0–3 min, 2% B; 6 min, 25% B; 10 min, 50% B; 14–17 min, 95% B; and 17.5 min, 2% B. The column temperature was maintained at 25 °C, the flow rate was 0.4 mL min^−1^, and the injection volume was 2 μL.

Mass spectrometric detection was performed using an electrospray ionization (ESI) source operated in both negative and positive ion modes under multiple reaction monitoring (MRM) conditions. The source parameters were as follows: capillary voltage, −3.5 kV; drying gas temperature, 300 °C; drying gas flow rate, 11 L min^−1^; and nebulizer pressure, 40 psi.

Analytes were identified by comparing their retention times and characteristic MRM transitions with those of authentic reference standards analyzed under identical chromatographic and mass spectrometric conditions. Quantification was performed using external calibration curves prepared from the corresponding authentic standards, and the results were expressed as μg/g extract. Compound-specific retention times, precursor and product ions, collision energies, calibration ranges, regression equations, coefficients of determination (R^2^), limits of detection (LOD), and limits of quantification (LOQ) are provided in Tables S1 and S2 of the Supplementary Materials.

### 4.4. Antioxidant Activity

The antioxidant properties of the organ extracts were evaluated using six complementary in vitro assays representing different chemical mechanisms. Phosphomolybdenum reducing capacity was determined according to Prieto et al. [27]. Radical-scavenging responses were assessed using the DPPH [28] and ABTS [29] assays, while ferrous-ion chelating activity was determined according to Dinis et al. [30]. Reducing capacity was further evaluated using the CUPRAC [31] and FRAP [32] assays. The assays were performed with minor modifications based on previously reported procedures [20,33,34]. Appropriate working concentrations were prepared from the 15 mg/mL methanolic stock solutions for each assay. Detailed reagent compositions, reaction volumes, incubation conditions, and spectrophotometric measurements are provided in the Supplementary Materials.

Antioxidant capacities were expressed as milligrams of Trolox equivalents per gram of extract (mg TE/g extract) for the phosphomolybdenum, DPPH, ABTS, CUPRAC, and FRAP assays and as milligrams of EDTA equivalents per gram of extract (mg EDTAE/g extract) for metal-chelating activity. Concentration-dependent responses were additionally evaluated using IC_50_ values for the DPPH and ABTS radical-scavenging assays and metal-chelating activity and EC_0.5_ values for the phosphomolybdenum, CUPRAC, and FRAP reducing-capacity assays. IC_50_ was defined as the extract concentration required to produce 50% inhibition, whereas EC_0.5_ was defined as the extract concentration required to produce an absorbance of 0.5 under the corresponding assay conditions. Both parameters were expressed as mg/mL, with lower values indicating greater activity.

### 4.5. Statistical and Multivariate Analyses

Each organ was represented by a single pooled composite sample, and a separate methanolic extract was prepared for each organ. All analytical measurements were performed in technical triplicate, and the results are expressed as mean ± standard deviation (SD).

Differences among the organ extracts were evaluated using one-way analysis of variance (ANOVA), followed by Tukey’s post hoc multiple-comparison test. This statistical procedure was applied to LC–ESI–MS/MS phenolic compound concentrations, total phenolic content (TPC), total flavonoid content (TFC), antioxidant equivalent values, IC_50_ and EC_0.5_ values, and Relative Antioxidant Capacity Index (RACI) values. Differences were considered statistically significant at *p* < 0.05. The triplicate measurements represented technical replicates of each organ-specific extract rather than independent biological replicates. Technical replication primarily captures measurement- and procedure-related variability and does not substitute for independent biological replication when estimating biological variability [35]. Accordingly, the ANOVA/Tukey comparisons reported herein should be interpreted as analytical-level comparisons among the investigated organ extracts and not as estimates of biological variability among independent plants or populations.

For the antioxidant assays, concentration-dependent data obtained at the tested extract concentrations were subjected to linear regression analysis using Microsoft Excel (Microsoft Corporation, Redmond, WA, USA). For the DPPH and ABTS radical-scavenging assays and metal-chelating activity, percentage inhibition values were regressed against extract concentration, and IC_50_ values were calculated as the extract concentrations required to produce 50% inhibition. For the phosphomolybdenum, CUPRAC, and FRAP reducing-capacity assays, absorbance values were regressed against extract concentration, and EC_0.5_ values were calculated from the resulting regression equations as the extract concentrations required to produce an absorbance of 0.5.

The Relative Antioxidant Capacity Index (RACI) was calculated according to Sun and Tanumihardjo [15]. For each of the three technical replicates, the individual values of TPC, TFC, and the six antioxidant equivalent parameters obtained from the phosphomolybdenum, DPPH, ABTS, CUPRAC, FRAP, and metal-chelating assays were standardized across the five organs as *z*-scores according to *z* = (*x* − μ)/σ, where *x* represents the individual value, μ is the mean, and σ is the standard deviation of the corresponding variable across the five organs. The standardized values were then averaged to obtain a RACI value for each technical replicate. The three resulting RACI values for each organ were expressed as mean ± SD and were compared among organs using one-way ANOVA followed by Tukey’s post hoc test. Higher RACI values indicate a comparatively stronger overall antioxidant profile across the variables included in the index.

Pearson correlation analysis was performed using organ-level mean values across the five organ profiles (*n* = 5). The correlation matrix comprised 28 variables: the 20 phenolic compounds detected by LC–ESI–MS/MS, TPC, TFC, and the six antioxidant equivalent values obtained from the phosphomolybdenum, DPPH, ABTS, CUPRAC, FRAP, and metal-chelating assays. For the multivariate analyses, phenolic compounds reported as not detected (nd) in individual organ extracts in Table 2 were assigned a value of zero prior to analysis. This zero value was used operationally to represent analytes not detected under the applied LC–ESI–MS/MS conditions and should not be interpreted as evidence of absolute absence from the plant material. Compounds that were not detected in any of the five organs were excluded from the multivariate dataset. Accordingly, the dataset comprised the 20 phenolic compounds detected in at least one organ, together with TPC, TFC, and the six antioxidant equivalent values, yielding a total of 28 variables. IC_50_ and EC_0.5_ values were not included in the correlation analysis. Pearson correlation coefficients (*r*) were used to describe the direction and magnitude of linear associations among the variables. Given the limited number of organ-level profiles, the correlations were interpreted primarily as exploratory associations rather than as evidence of causal relationships.

Hierarchical cluster analysis (HCA) was performed on the Pearson correlation matrix using the unweighted pair-group method with arithmetic mean (UPGMA; average linkage), with 1 − *r* used as the correlation-based distance measure. The resulting dendrogram was integrated with the Pearson correlation heatmap to visualize groups of variables exhibiting similar organ-dependent covariation patterns.

Principal component analysis (PCA) was performed on the same 28-variable dataset using the Pearson correlation matrix. PCA score plots were used to visualize the relative positioning of the five organ profiles, whereas variable loadings were examined to identify the phytochemical and antioxidant variables contributing most strongly to their distribution in multivariate space. The first two principal components were retained for graphical interpretation.

One-way ANOVA, Tukey’s post hoc test, Pearson correlation analysis, HCA, and PCA were performed using SPSS version 26.0 (IBM Corp., Armonk, NY, USA), whereas the linear regression analyses used for IC_50_ and EC_0.5_ calculations were conducted using Microsoft Excel.

## 5. Conclusions

This study provides an organ-resolved characterization of the phenolic composition and antioxidant responses of *C. cancellatus*. Targeted LC–ESI–MS/MS profiling revealed pronounced chemical differentiation among the stamen, pistil, tepal, perigon tube, and corm. The tepal was characterized by marked enrichment in hesperidin together with elevated hyperoside and gallic acid, whereas the pistil showed a distinct hydroxybenzoic- and hydroxycinnamic-acid-rich profile. The stamen was distinguished by the enrichment of protocatechuic acid, pyrocatechol, and verbascoside and by the detection of apigenin and taxifolin, while the corm was characterized by comparatively high pinoresinol and caffeic acid levels and the exclusive detection of vanillin. These findings demonstrate substantial organ-associated partitioning of phenolic constituents within the analyzed plant material.

Antioxidant responses were strongly assay-dependent, with no single organ consistently showing the highest response across all chemical mechanisms. The contrasting patterns observed among radical-scavenging, reducing-capacity, and metal-chelating assays indicate that antioxidant behavior cannot be adequately represented by a single assay or by total phenolic or flavonoid content alone. RACI provided a complementary integrated measure of the phytochemical and antioxidant variables and highlighted the pistil and stamen as the organs with the highest composite profiles.

Pearson correlation analysis, HCA, and PCA further demonstrated that the five organs were associated with distinct combinations of phenolic constituents and antioxidant variables. The multivariate patterns were consistent with the phenolic-acid-rich character of the pistil, the flavonoid-rich profile of the tepal, the catechol- and verbascoside-associated profile of the stamen, and the pinoresinol/caffeic-acid-associated profile of the corm. These relationships should be interpreted as patterns of organ-level co-variation rather than evidence of direct causal contributions of individual compounds to antioxidant activity.

Overall, the present findings establish a phytochemical baseline for the analyzed population of *C. cancellatus* and highlight the value of organ-resolved analysis for revealing chemical variation that may be obscured when plant material is examined as a whole. Validation across independent populations and collection periods, together with broader metabolomic profiling, isolation of selected constituents, and cellular antioxidant models, will be important for determining the reproducibility and biological relevance of the organ-associated signatures identified here.

## Figures and Tables

**Table 1:** Relative antioxidant capacity index (RACI), total phenolic content, and total flavonoid content of ultrasound-assisted methanolic extracts obtained from different organs of *C. cancellatus*.

Plant Organ	RACI	Total Phenolic Content (mg GAE/g Extract)	Total Flavonoid Content (mg RE/g Extract)
Stamen	0.38 ± 0.10	12.63 ± 0.05 ^a^	6.00 ± 2.94 ^d^
Pistil	0.52 ± 0.06	12.40 ± 0.19 ^a^	12.67 ± 0.19 ^c^
Tepal	0.16 ± 0.07	9.90 ± 0.19 ^b^	81.50 ± 0.40 ^a^
Perigon tube	−0.98 ± 0.03	5.37 ± 0.28 ^c^	18.92 ± 0.34 ^b^
Corm	−0.08 ± 0.07	11.71 ± 0.42 ^a^	0.63 ± 0.01 ^e^

Values are expressed as mean ± SD of technical triplicate measurements (*n* = 3 technical replicates) performed on a single organ-specific extract prepared from a pooled composite sample. Accordingly, ANOVA/Tukey comparisons reflect analytical-level differences based on technical replication and do not represent biological variability among independent plants or populations. Different superscript lowercase letters within the same column indicate statistically significant differences according to one-way analysis of variance (ANOVA) followed by Tukey’s honestly significant difference (HSD) post hoc test at the 5% significance level (*p* < 0.05). Total phenolic content (TPC) was expressed as mg gallic acid equivalents (GAE)/g extract, whereas total flavonoid content (TFC) was expressed as mg rutin equivalents (RE)/g extract. RACI values were calculated by standardizing TPC, TFC, and the six antioxidant equivalent values as z-scores and averaging the resulting standardized values for each organ. Higher RACI values indicate a comparatively stronger integrated profile across the variables included in the index.

**Table 2:** Phenolic compound profile of ultrasound-assisted methanolic extracts obtained from different organs of *C. cancellatus* determined by LC–MS/MS analysis.

No	Compounds	Stamen	Pistil	Tepal	Perigon Tube	Corm
1	Hesperidin	5479 ± 23 ^c^	1299 ± 8 ^d^	17660 ± 60 ^a^	8453 ± 27 ^b^	411 ± 6 ^e^
2	Hyperoside	106 ± 1 ^b^	129 ± 2 ^b^	286 ± 20 ^a^	60.6 ± 1.3 ^c^	1.55 ± 0.06 ^d^
3	Gallic acid	1.86 ± 0.02 ^d^	3.39 ± 0.12 ^c^	81.1 ± 0.4 ^a^	2.72 ± 0.06 ^c^	4.63 ± 0.01 ^b^
4	Quercetin	22.1 ± 0.1 ^d^	45.6 ± 0.3 ^a^	27.4 ± 0.2 ^c^	42.3 ± 0.1 ^b^	3.02 ± 0.02 ^e^
5	3-Hydroxybenzoic acid	106 ± 1 ^b^	331 ± 1 ^a^	16.6 ± 0.6 ^d^	51.7 ± 0.6 ^c^	16.7 ± 0.3 ^d^
6	4-Hydroxybenzoic acid	107 ± 2 ^b^	341 ± 2 ^a^	16.3 ± 0.1 ^d^	51.9 ± 0.4 ^c^	16.7 ± 0.2 ^d^
7	Apigenin 7-glucoside	14.5 ± 0.3 ^a^	nd	13.4 ± 0.6 ^a^	nd	nd
8	Pyrocatechol	19.4 ± 0.5 ^a^	18.1 ± 0.1 ^b^	1.40 ± 0.07 ^e^	4.51 ± 0.35 ^c^	2.65 ± 0.09 ^d^
9	Syringic acid	1.99 ± 0.45 ^cd^	11.0 ± 0.3 ^a^	1.36 ± 0.03 ^d^	4.92 ± 0.38 ^b^	3.04 ± 0.18 ^c^
10	Protocatechuic acid	20.9 ± 0.5 ^a^	16.9 ± 0.2 ^b^	0.73 ± 0.03 ^d^	3.33 ± 0.13 ^c^	3.21 ± 0.08 ^c^
11	Verbascoside	8.53 ± 0.04 ^a^	1.36 ± 0.03 ^c^	0.52 ± 0.01 ^d^	1.16 ± 0.02 ^c^	2.47 ± 0.28 ^b^
12	Caffeic acid	4.16 ± 0.03 ^b^	2.99 ± 0.04 ^c^	0.48 ± 0.01 ^d^	1.15 ± 0.02 ^d^	21.7 ± 0.6 ^a^
13	Ferulic acid	8.08 ± 0.26 ^c^	45.5 ± 0.1 ^a^	0.40 ± 0.02 ^e^	6.54 ± 0.47 ^d^	11.5 ± 0.1 ^b^
14	Sinapic acid	1.83 ± 0.05 ^bc^	14.8 ± 0.3 ^a^	0.40 ± 0.01 ^d^	2.17 ± 0.14 ^b^	1.43 ± 0.07 ^c^
15	Luteolin 7-glucoside	nd	nd	nd	nd	nd
16	Apigenin	13.6 ± 0.4	nd	nd	nd	nd
17	2,5-Dihydroxybenzoic acid	nd	nd	nd	nd	nd
18	3,4-Dihydroxyphenylacetic acid	nd	nd	nd	nd	nd
19	(+)-Catechin	nd	nd	nd	nd	nd
20	Chlorogenic acid	2.00 ± 0.30 ^b^	nd	nd	nd	4.10 ± 0.17 ^a^
21	(-)-Epicatechin	nd	nd	nd	nd	nd
22	Vanillin	nd	nd	nd	nd	16.0 ± 0.6
23	Taxifolin	2.96 ± 0.01	nd	nd	nd	nd
24	*p*-Coumaric acid	30.6 ± 0.1 ^b^	39.6 ± 0.7 ^a^	nd	10.2 ± 0.5 ^d^	28.3 ± 0.3 ^c^
25	Rosmarinic acid	nd	nd	nd	nd	nd
26	2-Hydroxycinnamic acid	nd	nd	nd	nd	nd
27	Pinoresinol	nd	nd	nd	1.69 ± 0.08 ^b^	60.1 ± 0.8 ^a^
28	Eriodictyol	nd	nd	nd	nd	nd
29	Luteolin	nd	nd	nd	nd	nd
30	Kaempferol	nd	nd	nd	nd	nd

Values are expressed as mean ± SD of technical triplicate measurements (*n* = 3 technical replicates) performed on a single organ-specific extract prepared from a pooled composite sample. Phenolic compound concentrations are expressed as μg/g extract. Accordingly, ANOVA/Tukey comparisons reflect analytical-level differences based on technical replication and do not represent biological variability among independent plants or populations. Different superscript lowercase letters within the same row indicate statistically significant analytical-level differences according to one-way ANOVA followed by Tukey’s HSD test (*p* < 0.05). nd, not detected under the applied LC–ESI–MS/MS conditions.

**Table 3:** Antioxidant capacities of ultrasound-assisted methanolic extracts obtained from different organs of *C. cancellatus*, expressed as Trolox equivalents (mg TE/g extract) and EDTA equivalents (mg EDTAE/g extract).

Assay	Stamen	Pistil	Tepal	Perigon Tube	Corm
Phosphomolybdenum (mg TE/g extract)	376.84 ± 29.33 ^a^	279.31 ± 13.73 ^b^	394.93 ± 33.70 ^a^	195.47 ± 11.23 ^b^	252.39 ± 10.61 ^b^
DPPH (mg TE/g extract)	12.48 ± 0.24 ^c^	26.55 ± 0.72 ^b^	29.87 ± 1.59 ^ab^	15.12 ± 4.46 ^c^	35.38 ± 0.64 ^a^
ABTS (mg TE/g extract)	35.29 ± 1.22 ^d^	58.12 ± 0.06 ^a^	39.00 ± 1.11 ^c^	28.49 ± 0.70 ^e^	46.17 ± 0.64 ^b^
CUPRAC (mg TE/g extract)	71.04 ± 1.44 ^a^	42.99 ± 2.20 ^b^	35.07 ± 3.37 ^b^	19.42 ± 3.23 ^c^	37.60 ± 2.54 ^b^
FRAP (mg TE/g extract)	37.66 ± 0.89 ^b^	43.77 ± 0.32 ^a^	17.42 ± 0.16 ^e^	21.81 ± 1.37 ^d^	28.65 ± 0.56 ^c^
Metal chelation (mg EDTAE/g extract)	16.29 ± 0.08 ^a^	16.21 ± 0.07 ^ab^	15.99 ± 0.10 ^ab^	15.98 ± 0.06 ^ab^	15.87 ± 0.14 ^b^

Values are expressed as mean ± SD of technical triplicate measurements (*n* = 3 technical replicates) performed on a single organ-specific extract prepared from a pooled composite sample. Accordingly, ANOVA/Tukey comparisons reflect analytical-level differences based on technical replication and do not represent biological variability among independent plants or populations. Different superscript lowercase letters within the same row indicate statistically significant differences according to one-way analysis of variance (ANOVA) followed by Tukey’s honestly significant difference (HSD) post hoc test at the 5% significance level (*p* < 0.05). Trolox equivalent (TE) was used as the reference standard for the phosphomolybdenum, DPPH, ABTS, CUPRAC, and FRAP assays. EDTA equivalent (EDTAE) was used for the metal-chelating activity assay. All results are expressed on an extract dry weight basis (g extract).

**Table 4:** Inhibitory concentrations (IC_50_) and concentrations producing an absorbance of 0.5 (EC_0.5_), expressed in mg/mL, of ultrasound-assisted methanolic extracts obtained from different organs of *C. cancellatus* and the corresponding reference standards.

Assay	Stamen	Pistil	Tepal	Perigon Tube	Corm	Standard
Phosphomolybdenum (EC_0.5_)	1.62 ± 0.13 ^b^	2.19 ± 0.11 ^c^	1.55 ± 0.13 ^b^	3.13 ± 0.18 ^d^	2.42 ± 0.10 ^c^	0.61 ± 0.01 ^a^
DPPH (IC_50_)	11.22 ± 0.21 ^e^	5.28 ± 0.14 ^cd^	4.69 ± 0.25 ^bc^	9.68 ± 2.85 ^de^	3.96 ± 0.07 ^bc^	0.15 ± 0.01 ^a^
ABTS (IC_50_)	3.12 ± 0.11 ^e^	1.89 ± 0.01 ^b^	2.82 ± 0.08 ^d^	3.86 ± 0.09 ^f^	2.38 ± 0.03 ^c^	0.11 ± 0.01 ^a^
CUPRAC (EC_0.5_)	1.32 ± 0.03 ^bc^	2.19 ± 0.11 ^c^	2.69 ± 0.26 ^c^	4.91 ± 0.82 ^d^	2.51 ± 0.17 ^c^	0.094 ± 0.001 ^a^
FRAP (EC_0.5_)	2.52 ± 0.06 ^b^	2.17 ± 0.02 ^b^	5.45 ± 0.05 ^e^	4.36 ± 0.27 ^d^	3.32 ± 0.07 ^c^	0.095 ± 0.001 ^a^
Metal chelation (IC_50_)	1.04 ± 0.01 ^b^	1.050 ± 0.004 ^b^	1.06 ± 0.01 ^bc^	1.06 ± 0.01 ^bc^	1.07 ± 0.01 ^c^	0.017 ± 0.001 ^a^

Values are expressed as mean ± SD of technical triplicate measurements (*n* = 3 technical replicates) performed on a single organ-specific extract prepared from a pooled composite sample. Accordingly, ANOVA/Tukey comparisons reflect analytical-level differences based on technical replication and do not represent biological variability among independent plants or populations. IC_50_ was defined as the extract concentration required to produce 50% inhibition in the DPPH, ABTS, and metal-chelating assays, whereas EC_0.5_ was defined as the extract concentration required to produce an absorbance of 0.5 in the phosphomolybdenum, CUPRAC, and FRAP assays. Both IC_50_ and EC_0.5_ values are expressed as mg/mL, with lower values indicating greater activity under the applied assay conditions. Trolox was used as the reference standard for the phosphomolybdenum, DPPH, ABTS, CUPRAC, and FRAP assays, whereas EDTA was used for the metal-chelating assay. Different superscript lowercase letters within the same row indicate statistically significant differences.

## Data Availability

The original contributions presented in this study are included in the article/Supplementary Material. Further inquiries can be directed to the corresponding author.

## Supplementary Materials

The following supporting information can be downloaded at: https://www.mdpi.com/article/10.3390/ijms27198904/s1.
