# Supplementary material for: Organ-Specific Phenolic Specialization and Antioxidant Responses in Crocus cancellatus: LC–MS/MS and Multivariate Analysis
Source: Int J Mol Sci. 2026 Oct 7;27(19):8904. doi: 10.3390/ijms27198904 (PMC13664975; doi:10.3390/ijms27198904)
Supplement: Supplementary file 1 [file ijms-27-08904-s001.zip › ijms-4566537-supplementary.pdf]

Supplementary Materials

1. Chemicals

Gallic acid (97.5–102.5%, titration), (+)-catechin (≥99.0%, HPLC), pyrocatechol (≥99%), chlorogenic acid (≥95%, titration), 2,5-dihydroxybenzoic acid (≥99.0%, HPLC), 4-hydroxybenzoic acid (≥99%), (−)-epicatechin (≥97.0%, HPLC), caffeic acid (≥98.0%, HPLC), syringic acid (≥98.0%, HPLC), vanillin (99%), (+)-taxifolin (≥95.0%, HPLC), sinapic acid (≥98%), p-coumaric acid (≥98.0%, HPLC), ferulic acid (≥99.0%, HPLC), rosmarinic acid (96%), 2-hydroxycinnamic acid (97%), pinoresinol (≥95.0%, HPLC), quercetin (≥95%, HPLC), luteolin (≥97.0%, HPLC), apigenin (≥99%, HPLC), protocatechuic acid (3,4-dihydroxybenzoic acid; ≥97.0%, titration), and verbascoside (≥99%, HPLC) were purchased from Sigma-Aldrich (St. Louis, MO, USA).

3-Hydroxybenzoic acid (≥99.0%, HPLC), 3,4-dihydroxyphenylacetic acid (≥97.0%, HPLC), apigenin 7-glucoside (≥97.0%, HPLC), luteolin 7-glucoside (≥98.0%, HPLC), hesperidin (≥97.0%, HPLC), eriodictyol (≥98.0%, HPLC), and kaempferol (≥99.0%, HPLC) were obtained from Fluka (St. Louis, MO, USA). Hyperoside was purchased from HWI Analytik (Ruelzheim, Germany) as a primary reference standard, with the exact content specified in the corresponding lot-specific certificate of analysis. HPLC-grade methanol and formic acid were purchased from Sigma-Aldrich (St. Louis, MO, USA) and Merck (Darmstadt, Germany), respectively. Ultrapure water was obtained using a Milli-Q Plus water purification system (Millipore Bedford Corp., Bedford, MA, USA). Unless otherwise specified, all other reagents and chemicals were of analytical grade or higher.

2. Phytochemical analysis

2.1. Determination of Total Phenolic and Flavonoid Contents

Total phenolic content (TPC) was determined using the Folin–Ciocalteu method. Briefly, 0.25 mL of the sample solution was mixed with 1.0 mL of Folin–Ciocalteu reagent diluted 1:9 (v/v) with water and mixed thoroughly. After 3 min, 0.75 mL of 1% (w/v) Na_2CO_3 solution was added. The reaction mixture was incubated for 2 h at room temperature, and absorbance was measured at 760 nm. TPC was expressed as milligrams of gallic acid equivalents per gram of extract (mg GAE/g extract).

Total flavonoid content (TFC) was determined using the aluminium chloride colorimetric method. Sample solution (1.0 mL) was mixed with 1.0 mL of 2% (w/v) aluminium chloride in methanol. A corresponding blank was prepared by mixing 1.0 mL of sample solution with 1.0 mL of methanol without aluminium chloride. Following incubation for 10 min at room temperature, absorbance was measured at 415 nm. The absorbance of the corresponding blank was subtracted from that of the sample. TFC was expressed as milligrams of rutin equivalents per gram of extract (mg RE/g extract).

2.2. LC–ESI–MS/MS Analysis of Phenolic Compounds

Targeted phenolic analysis was performed using an LC–ESI–MS/MS method adapted from the previously validated procedure reported by [26]. The analytical panel comprised 30 phenolic compounds. Quantitative analyses were performed using an Agilent Technologies 1260 Infinity liquid chromatography system coupled to an Agilent 6420 Triple Quadrupole mass spectrometer. Chromatographic separation was achieved on a Poroshell 120 EC-C18 column (100 mm × 4.6 mm i.d., 2.7 μm particle size).

The mobile phase consisted of solvent A (0.1% v/v formic acid in water) and solvent B (methanol). The gradient program was as follows: 0–3 min, 2% B; 6 min, 25% B; 10 min, 50% B; 14–17 min, 95% B; and

17.5 min, 2% B. The column temperature was maintained at 25 °C, the flow rate was 0.4 mL min^{-1}, and the injection volume was 2 μL.

The tandem mass spectrometer was operated with an electrospray ionization (ESI) source in both negative and positive ionization modes using multiple reaction monitoring (MRM). The ESI source parameters were as follows: capillary voltage, −3.5 kV; drying gas temperature, 300 °C; drying gas flow rate, 11 L min^{-1}; and nebulizer pressure, 40 psi.

Analytes were identified by comparing their retention times and characteristic MRM transitions with those of authentic reference standards analyzed under identical chromatographic and mass spectrometric conditions. Quantification was performed using external calibration curves prepared from the corresponding authentic standards. Quantitative results were expressed as micrograms of compound per gram of extract (μg/g extract). Compound-specific retention times, precursor ions, product-ion transitions, and collision energies are presented in Table S1, while calibration ranges, regression equations, coefficients of determination (R^2), limits of detection (LOD), and limits of quantification (LOQ) are summarized in Table S2.

Supplementary Table S1. LC–ESI–MS/MS parameters and analytical characteristics of the target phenolic compounds analyzed by multiple reaction monitoring in negative and positive ionization modes

Target compounds	Rt (min)	Precursor ion	MRM1 (CE, V)	MRM2 (CE, V)
Compounds analyzed by NI mode				
Gallic acid	8.891	168.9 [M − H]−	125.0 (10)	–
Protocatechuic acid	10.818	152.9 [M − H]−	108.9 (12)	–
3,4-Dihydroxyphenylacetic acid	11.224	167.0 [M − H]−	123.0 (2)	–
(+)-Catechin	11.369	289.0 [M − H]−	245.0 (6)	202.9 (12)
Pyrocatechol	11.506	109.0 [M − H]−	90.6 (18)	52.9 (16)
2,5-Dihydroxybenzoic acid	12.412	152.9 [M − H]−	109.0 (10)	–
4-Hydroxybenzoic acid	12.439	136.9 [M − H]−	93.1 (14)	–
Caffeic acid	12.841	179.0 [M − H]−	135.0 (12)	–
Syringic acid	12.963	196.9 [M − H]−	181.9 (8)	152.8 (6)
3-Hydroxybenzoic acid	13.259	137.0 [M − H]−	93.0 (6)	–
Vanillin	13.397	151.0 [M − H]−	136.0 (10)	–
Verbascoside	13.589	623.0 [M − H]−	461.0 (26)	160.8 (36)
Taxifolin	13.909	303.0 [M − H]−	285.1 (2)	125.0 (14)
Sinapic acid	13.992	222.9 [M − H]−	207.9 (6)	163.8 (6)
p-Coumaric acid	14.022	162.9 [M − H]−	119.0 (12)	–
Ferulic acid	14.120	193.0 [M − H]−	177.8 (8)	134.0 (12)
Luteolin 7-glucoside	14.266	447.1 [M − H]−	285.0 (24)	–
Rosmarinic acid	14.600	359.0 [M − H]−	196.9 (10)	160.9 (10)
2-Hydroxycinnamic acid	15.031	162.9 [M − H]−	119.1 (10)	–
Pinoresinol	15.118	357.0 [M − H]−	151.0 (12)	135.7 (34)
Eriodictyol	15.247	287.0 [M − H]−	151.0 (4)	134.9 (22)
Quercetin	15.668	301.0 [M − H]−	178.6 (10)	151.0 (16)
Kaempferol	16.236	285.0 [M − H]−	242.8 (16)	229.1 (18)
Compounds analyzed by PI mode				
Chlorogenic acid	11.802	355.0 [M + H]+	163.0 (10)	–
(−)-Epicatechin	12.458	291.0 [M + H]+	139.1 (12)	122.9 (36)
Hesperidin	14.412	611.1 [M + H]+	449.2 (4)	303.0 (20)
Hyperoside	14.506	465.1 [M + H]+	303.1 (8)	–
Apigenin 7-glucoside	14.781	433.1 [M + H]+	271.0 (18)	–

Luteolin	15.923	287.0 [M + H]+	153.1 (34)	135.1 (36)
Apigenin	16.382	271.0 [M + H]+	153.0 (34)	119.1 (36)

Rt, retention time; NI, negative ionization mode; PI, positive ionization mode; MRM, multiple reaction monitoring; CE, collision energy.

Supplementary Table S2. Calibration curves, linearity, and sensitivity characteristics of the LC–ESI–MS/MS method

Compounds	Linearity and sensitivity characteristics				
	Range (µg/L)	Linear equation	R^2	LOD (µg/L)	LOQ (µg/L)
Gallic acid	5–500	y = 4.82x − 26.48	0.9988	1.46	4.88
Protocatechuic acid	2.5–500	y = 5.65x − 9.99	0.9990	1.17	3.88
3,4-Dihydroxyphenylacetic acid	5–500	y = 5.13x − 12.39	0.9990	1.35	4.51
(+)-Catechin	10–500	y = 1.45x + 1.95	0.9974	3.96	13.20
Pyrocatechol	25–400	y = 0.11x − 0.52	0.9916	9.62	32.08
Chlorogenic acid	1–500	y = 12.14x + 32.34	0.9995	0.55	1.82
2,5-Dihydroxybenzoic acid	5–500	y = 3.79x − 14.12	0.9980	2.12	7.08
4-Hydroxybenzoic acid	5–500	y = 7.62x + 22.79	0.9996	1.72	5.72
(−)-Epicatechin	5–500	y = 9.11x − 9.99	0.9971	1.85	6.18
Caffeic acid	5–500	y = 11.09x + 16.73	0.9997	3.15	10.50
Syringic acid	10–500	y = 0.74x − 1.54	0.9975	3.75	12.50
3-Hydroxybenzoic acid	5–500	y = 3.69x − 12.29	0.9991	1.86	6.20
Vanillin	50–500	y = 2.02x + 135.49	0.9926	15.23	50.77
Verbascoside	2.5–500	y = 8.59x − 28.05	0.9988	0.82	2.75
Taxifolin	5–500	y = 12.32x + 9.98	0.9993	1.82	6.05
Sinapic acid	5–500	y = 2.09x − 6.79	0.9974	2.64	8.78
p-Coumaric acid	5–500	y = 17.51x + 53.73	0.9997	1.93	6.44
Ferulic acid	5–500	y = 3.32x − 4.30	0.9992	1.43	4.76
Luteolin 7-glucoside	1–500	y = 45.25x + 156.48	0.9996	0.45	1.51
Hesperidin	5–500	y = 5.98x + 0.42	0.9993	1.73	5.77
Hyperoside	2.5–500	y = 16.32x − 1.26	0.9998	0.99	3.31
Rosmarinic acid	1–500	y = 9.82x − 17.98	0.9989	0.57	1.89
Apigenin 7-glucoside	1–500	y = 21.33x − 31.69	0.9983	0.41	1.35
2-Hydroxycinnamic acid	1–500	y = 16.72x − 26.94	0.9996	0.61	2.03
Pinoresinol	10–500	y = 0.80x − 2.69	0.9966	3.94	13.12
Eriodictyol	2.5–500	y = 14.24x − 0.50	0.9998	0.80	2.68
Quercetin	5–500	y = 14.68x − 18.25	0.9997	1.23	4.10
Luteolin	5–500	y = 8.96x + 26.80	0.9992	1.34	4.46
Kaempferol	10–500	y = 0.82x − 3.06	0.9959	3.30	10.99
Apigenin	2.5–500	y = 11.29x + 38.05	0.9987	0.96	3.20

R^2, coefficient of determination; LOD, limit of detection; LOQ, limit of quantification.

3. Antioxidant Activity

3.1. Preparation of Extract Solutions

For antioxidant assays, each methanolic extract was redissolved in methanol to obtain a stock solution at a concentration of 15 mg/mL. Appropriate working concentrations were subsequently prepared by dilution of the stock solutions according to the requirements of each assay.

3.2. Phosphomolybdenum Assay

Total reducing capacity was evaluated using the phosphomolybdenum assay. Sample solution (0.2 mL) was mixed with 2.0 mL of reagent solution containing 0.6 M sulfuric acid, 28 mM sodium phosphate, and 4 mM ammonium molybdate. The reaction mixture was incubated at 95 °C for 90 min, and absorbance was subsequently measured at 695 nm.

3.3. DPPH Radical Scavenging Assay

DPPH radical scavenging activity was determined by mixing 1.0 mL of sample solution with 4.0 mL of a 0.004% (w/v) methanolic DPPH solution. The reaction mixture was incubated for 30 min at room temperature in the dark, after which absorbance was measured at 517 nm.

3.4. ABTS Radical Cation Scavenging Assay

The ABTS radical cation ($ABTS^{\bullet+}$) was generated by reacting 7 mM ABTS with 2.45 mM potassium persulfate and allowing the mixture to stand for 12–16 h at room temperature in the dark. Before use, the $ABTS^{\bullet+}$ solution was diluted with methanol to obtain an absorbance of 0.700 ± 0.020 at 734 nm. Sample solution (1.0 mL) was mixed with 2.0 mL of the diluted $ABTS^{\bullet+}$ solution, and absorbance was measured at 734 nm after 7 min of incubation at room temperature.

3.5. Metal Chelating Activity

Ferrous-ion chelating activity was determined by mixing 2.0 mL of sample solution with 0.05 mL of 2 mM $FeCl_2$ solution. The reaction was initiated by adding 0.2 mL of 5 mM ferrozine. A corresponding blank was prepared using water instead of ferrozine. After incubation for 10 min at room temperature, absorbance was measured at 562 nm.

3.6. CUPRAC Assay

Cupric reducing antioxidant capacity (CUPRAC) was determined by mixing 0.5 mL of sample solution with a reaction mixture containing 1.0 mL of 10 mM $CuCl_2$, 1.0 mL of 7.5 mM neocuproine, and 1.0 mL of 1 M ammonium acetate buffer (pH 7.0). A corresponding blank was prepared without $CuCl_2$. Following incubation for 30 min at room temperature, absorbance was measured at 450 nm.

3.7. FRAP Assay

Ferric reducing antioxidant power (FRAP) was evaluated using a freshly prepared reagent consisting of 0.3 M acetate buffer (pH 3.6), 10 mM 2,4,6-tris(2-pyridyl)-s-triazine (TPTZ) prepared in 40 mM HCl, and 20 mM $FeCl_3$ in a ratio of 10:1:1 (v/v/v). Sample solution (0.1 mL) was added to 2.0 mL of FRAP reagent, and absorbance was measured at 593 nm after 30 min of incubation at room temperature.

3.8. Expression of Antioxidant Results

Antioxidant responses were evaluated over appropriate concentration ranges prepared from the 15 mg/mL stock solutions. For the DPPH and ABTS radical-scavenging assays and ferrous-ion chelating activity, percentage inhibition values were plotted against extract concentration, and IC_{50} values were determined by linear regression as the extract concentrations required to produce 50% inhibition. For the phosphomolybdenum, CUPRAC, and FRAP reducing-capacity assays, absorbance values were plotted against extract concentration, and $EC_{0.5}$ values were determined from the corresponding linear regression equations as the extract concentrations required to produce an absorbance of 0.5. IC_{50} and $EC_{0.5}$ values were expressed as mg/mL, with lower values indicating greater activity under the applied assay conditions. Antioxidant capacities were additionally expressed as milligrams of standard

equivalent per gram of extract. Trolox was used as the reference standard for the phosphomolybdenum, DPPH, ABTS, CUPRAC, and FRAP assays, whereas disodium ethylenediaminetetraacetate (EDTA) was used as the reference standard for metal-chelating activity. Accordingly, results were expressed as mg TE/g extract or mg EDTAE/g extract, respectively.
